# Vitamin D Receptor Activation Influences NADPH Oxidase (NOX_2_) Activity and Protects against Neurological Deficits and Apoptosis in a Rat Model of Traumatic Brain Injury

**DOI:** 10.1155/2017/9245702

**Published:** 2017-12-19

**Authors:** Changmeng Cui, Sixin Song, Jianzhong Cui, Yan Feng, Junling Gao, Pei Jiang

**Affiliations:** ^1^Department of Neurosurgery, Affiliated Hospital of Jining Medical University, Jining, Shandong 272000, China; ^2^School of Basic Medical Science, North China University of Science and Technology, Tangshan, Hebei 063000, China; ^3^Department of Neurosurgery, Tangshan Gongren Hospital, Tangshan, Hebei 063000, China; ^4^Department of Neurosurgery, Affiliated Hospital of Taishan Medical University, Taian, Shandong 271000, China; ^5^Department of Neurosurgery, Second Hospital of Hebei Medical University, Shijiazhuang, Hebei 050000, China; ^6^Institute of Clinical Pharmacy and Pharmacology, Jining First People's Hospital, Jining Medical University, Jining, Shandong 272000, China

## Abstract

Traumatic brain injury (TBI) is a worldwide phenomenon which results in significant neurological and cognitive deficits in humans. Vitamin D (VD) is implicated as a therapeutic strategy for various neurological diseases now. Recently, inhibition of the NADPH oxidase (NOX_2_) was reported to protect against oxidative stress (ROS) production. However, whether alterations in NOX_2_ expression and NOX activity are associated with calcitriol (active metabolite of VD) treatment following TBI remains unclear. In the present study, rats were randomly assigned to the sham, TBI, and calcitriol-treated groups. Calcitriol was administered intraperitoneally (2 *μ*g/kg) at 30 min, 24 h, and 48 h after TBI insult. We observed that calcitriol treatment alleviated neurobehavioral deficits and brain edema following TBI. At the molecular levels, administration of calcitriol activated the expression of VDR and downregulated NOX_2_ as well as suppressed apoptosis cell rate in the hippocampus CA1 region of TBI rats. In conclusion, our findings indicate that the protective effects of calcitriol may be related to the modulation of NADPH oxidase and thereby ultimately inhibited the progression of apoptosis. Calcitriol may be promising as a protective intervention following TBI, and more study is warranted for its clinical testing in the future.

## 1. Introduction

Traumatic brain injury (TBI) is a worldwide phenomenon that affects all ages and socioeconomic classes and results in variation of immediate and delayed motor and cognitive deficiencies [1, 2]. As patients often present with a complexity of lesions of various severity and regional distributions, the pathogenesis of TBI is incompletely understood [3]. TBI is caused by both primary and secondary injury mechanisms. Primary damage is due to mechanical factors and occurs immediately at the moment of injury. It takes the form of intracranial hemorrhage, diffuse axonal injury (DAI), and surface contusions [4]. In comparison, the secondary injury is delayed and is produced via complicating processes that are initiated at the moment of impact but do not present clinically for a period of hours to days following trauma. It includes damage due to brain edema, ischemia, and alterations in neuronal function [4]. Until now, patients are still inadequately treated because of the lack of effective therapies on TBI [5]. As a result, to find new effective therapeutic drugs or to develop novel therapeutic strategies is an important issue.

Nicotinamide adenine dinucleotide phosphate oxidase (NADPH oxidase) is a multiunit enzyme composed of several subunits that include several isoforms of NOX_1–5_ [6]. NOX_2_, a catalytic subunit of NOX, is localized to the cerebral cortex and hippocampus CA1 region. Experimental evidence suggested that overactivated NOX_2_ significantly contributed to oxidative damage to neurons in ischemic and traumatic conditions [7, 8]. Recently, inhibition of the NADPH oxidase complex was reported to protect against oxidative stress (ROS) production, blood-brain barrier disruption, and neuronal death in vivo [9]. Our previous research also demonstrated that treatment with NADPH oxidase complex inhibitor attenuated the expression and activation of the NOX_2_ protein and reduced brain edema and spatial learning deficits in TBI rats [8]. Vitamin D (VD), which is most commonly associated with the regulation of calcium homeostasis, is implicated as a pleiotropic secosteroid affecting multiple aspects of human physiology now [10]. The biologically active metabolite of VD (calcitriol, 1,25-dihydroxyvitamin D) exerts its endocrinological influence via a nuclear vitamin D receptor (VDR). The wide distribution of VDR suggests that VD may regulate various physiological pathways, such as brain development, cell cycle control, and immune modulation [11–15]. Moreover, emerging evidence also suggested that VD is developing as a therapeutic strategy for various neurological diseases, including depression, Parkinson's disease, epilepsy, and traumatic brain injury [16–19]. Our previous data showed the wide-ranged effects of calcitriol in the major neurotransmitter systems, providing new evidence for the role of VD in brain function [20]. However, whether alterations in NOX_2_ expression and NOX activity are associated with calcitriol treatment following TBI remains unclear.

In this experiment, through the treatment of calcitriol continuity in a rat model of TBI, we sought to assess the protective effect of ectogenic VD on TBI-induced neurological impairment and brain edema. We further examined whether the activation of VDR could attenuate neuron damage via modulation of the NADPH oxidase and cell apoptosis in the hippocampus CA1 region following TBI in rats.

## 2. Materials and Methods

### 2.1. Animals and TBI Model

Adult male Sprague-Dawley rats (age 10–12 weeks; weight 300–330 g; Tangshan, China) were used in this study. The Institutional Animal Care and Use Committee of North China University of Science and Technology approved all experiments, which were performed according to the guidelines of the National Institutes of Health Guide for the Care and Use of Laboratory Animals (NIH publication number 80-23, revised 1978). The rats were housed under environmentally controlled conditions in a 12 h light/dark cycle at 25°C and were provided with food and water. Efforts were made to reduce animal suffering and minimize the number of animals used for these experiments. A previously described TBI model was utilized [21]. Briefly, after inducing anesthesia with an intraperitoneal injection of 10% chloral hydrate (3 ml/kg), the head of the animal was fixed on a stereotactic frame. Aseptic techniques were used throughout the surgery. A midline scalp incision was performed to expose the skull. A 6 mm craniotomy was performed over the right parietal cortex, centered on the coronal suture and 2.5 mm lateral to the sagittal suture (velocity = 5 m/s, depth = 2.5 mm, and dwell time = 100 ms). The bone flap was immediately replaced and sealed, and the scalp was sutured closed. The rectal temperature was maintained at 37°C with heating pads and lamps. The animals were returned to the feeding room after recovery from anesthesia. Sham-operated rats underwent procedures identical to those of the TBI animals, including anesthesia and surgery, but without TBI.

### 2.2. Groups and Drug Administration

The 160 adult rats were each randomly assigned to one of the three groups: sham-operated (*n* = 40, sham), TBI model (*n* = 60, TBI), TBI + calcitriol treatment (*n* = 60, calcitriol), or sham + calcitriol (*n* = 15) treatment groups. Calcitriol (Sigma, USA, dissolved in 0.9% saline solution) was administered intraperitoneally (2 *μ*g/kg) at 30 min, 24 h, and 48 h after TBI insult. The dose was chosen based on previous findings showing the neuroprotective effects of calcitriol in the animal models of ischemia/reperfusion (I/R) injury and TBI [22, 23]. Both sham and TBI groups received equal volumes of saline by intraperitoneal injection. All investigations were blind, and the animal codes were revealed only at the end of the behavioral and histological analyses.

### 2.3. Evaluation of Neurological Scores

At 12 h and 1, 3, 7, and 14 days following TBI, the neurological functions were determined by neurological severity scores as previously described [24], a composite of motor, sensory, reflex, and balance tests (normal score: 2-3; maximal deficit score: 18).

### 2.4. Evaluation of Brain Edema

Brain edema was evaluated by the analysis of brain water content with the wet-dry weight method as described previously [8]. At 3 days following TBI or sham operation, rat brains were separated and weighed immediately with a chemical balance to get wet weight (WW). Following drying in a desiccating oven for 24 h at 100°C, dry tissues were weighed again to get constant dry weight (DW). The percentage of water in the tissues was calculated according to the following formula: %brain water = ((WW − DW)/WW) × 100.

### 2.5. Morris Water Maze (MWM) Test

The hippocampus-dependent spatial learning and memory was assessed in a MWM test at 7–10 days following TBI as our previous study [24]. Prior to operation, all rats were trained to find the platform. For each trial, the rat was randomly placed into a quadrant start point (N, S, E, or W) facing the wall of the pool and was allowed a maximum of 60 seconds to escape to the platform. If the rats failed to find the platform within 90 seconds, they were gently guided to the platform for a maximum of 20 seconds. Maze performance was recorded using a video camera suspended above the maze and interfaced with a video tracking system (HVS Imaging, Hampton, UK). The average escape latency of a total of four trials was recorded. On the final day, rats were subjected to a space exploring test, in which the platform was removed. Animals spent percentage of time in the target quadrant, and swim speeds were all evaluated in this test.

### 2.6. Immunohistochemistry (IHC) Staining

IHC staining was evaluated at 3 days after TBI or sham operation. After perfusion, brain tissues were fixed in 4% paraformaldehyde solution for 24 h, washed with running water for 4 h, then dehydrated with graded alcohol, and embedded in paraffin following standard histological procedures. Formalin-fixed paraffin-embedded sections (5 *μ*m) were blocked with 3% H_2_O_2_ for 20 min, followed by incubation with blocking 5% goat serum for 1 h at room temperature. The sections were then incubated with the NOX_2_ primary antibodies (1 : 500 diluted, Santa Cruz, CA, USA) overnight at 4°C, followed by incubation with secondary biotinylated antibodies (1 : 500 diluted, Santa Cruz, CA, USA) for 1 h. Color was developed with DAB reagent for 2–10 min. Images were captured using an AxioVision4Ac microscope system (Carl Zeiss, Germany).

### 2.7. NOX Activity Assay

The tissue samples of the hippocampal CA1 region were collected at 1, 3, and 7 days post-TBI. NOX activity analysis was performed as our previous study [8]. 50 *μ*g membrane fractions were used for assaying NOX enzymatic activity. Relative light units (RLU) were measured every minute continuously for 5 min via a standard luminometer. The results of NOX activity were calculated as RLU/*μ*g/minute.

### 2.8. Double Immunofluorescent Staining

Double immunofluorescent staining was evaluated at 3 days after TBI or sham operation. After perfusion, brains were removed, post-fixed in the same fixative for 1 day at room temperature, and subsequently soaked in 30% sucrose for 2-3 days. After that, the tissues were embedded in optimal cutting temperature (OCT) compound. Then, 12 *μ*m frozen cross sections were prepared and examined. The sections were incubated with mouse polyclonal primary antibodies for NeuN (a marker of neuron, 1 : 200 diluted, Sigma), GFAP (a marker of glial cells, 1 : 200 diluted, Santa Cruz, CA, USA), and rabbit polyclonal primary antibodies for NOX_2_ (1 : 200 diluted, Santa Cruz, CA, USA), respectively. They were incubated with all primary antibodies overnight at 4°C, followed by a mixture of FITC and TRITC-conjugated secondary antibodies for 2 h at room temperature. After washing with PBS 3 times for 10 min each, the sections were observed with a fluorescence microscope (Olympus Fluoview™ FV1000; Olympus, Tokyo, Japan).

### 2.9. Quantification of Confocal Images

The intensity of all confocal images was quantified using MATLAB software (version R2013a by Mathworks, Natick, MA, USA) as described previously [25]. MATLAB is a programming environment with built-in image processing tools. The intensity threshold for injured animals was identified by applying a multilevel image threshold algorithm using Otsu's method in MATLAB [26]. This value was then used as an intensity threshold for sham-operated and TBI animals. The algorithm digitized each image into a 1024 × 1024 matrix. The individual values contained in the matrix represented the intensity value of pixels of a particular color, that is, red or green. Using the threshold value obtained from the algorithm, the image was segmented into two regions: one above the threshold value and one below. Finally, dividing the segmented area with intensity above the threshold value by the total image area enabled image quantification. The data were obtained as the relative area of fluorescence as compared to the entire area of the image. The data was expressed as the percentage of area activated in the entire captured field.

### 2.10. Assessment of Apoptosis

The presence of apoptosis in the CA1 region of rat hippocampus was assessed by the terminal deoxynucleotidyl transferase-mediated FITC-dUTP nick end labeling (TUNEL) method following the manufacturer's protocol. Nuclei were counterstained with DAPI. The number of TUNEL-DAPI-positive cells was counted as described previously [27]. The counting area was located in the same position in all groups. For each group, quantification was performed in sections from three different rats.

### 2.11. Cell Culture and Stimulation

The HT22 cell line was a gift from North China University of Science and Technology (Tangshan, China). Cells were cultured in Dulbecco's modified Eagle's medium (DMEM) with 10% fetal bovine serum (FBS) and 1% penicillin/streptomycin at 37°C in a humidified atmosphere containing 95% oxygen and 5% CO_2_. We changed the medium every 2 days. The HT22 cells were assigned into 4 groups, including control, control + H_2_O_2_, calcitriol, and calcitriol + H_2_O_2._ HT22 cells were exposed to 100 nM calcitriol for 3 h after the cell density reached roughly 70–80%. In order to study oxidative stress in vitro, the control + H_2_O_2_ and calcitriol + H_2_O_2_ groups were exposed to concentrations of 200 *μ*M H_2_O_2_ for 3 h, which could decrease the cell metabolic activity by roughly 50% in a previous study [28].

### 2.12. Western Blot Analysis

Total protein extracts were obtained as described previously [29], separated by SDS–PAGE, transferred to PVDF, and immunoblotted using specific antibodies against VDR, NOX_2_, cleaved caspase-3, Bcl-2, and *β*-actin (1 : 1000 diluted, Santa Cruz, CA, USA). The membranes were incubated with secondary antibodies (1 : 3000 diluted, Santa Cruz, CA, USA) the next day. The immunoblotted proteins on the membrane were visualized following development with an enhanced chemiluminescence (ECL) detection system, and the densitometric signals were quantified by using ImageJ software (Image Lab 4.1; Bio-Rad).

### 2.13. Statistical Analysis

Data are expressed as the means ± standard error. All tests were performed using SPSS 17.0 software. Statistical significance was determined using one-way analysis of variance (ANOVA), and the Student-Newman-Keuls post hoc test was used to determine differences among different groups. *P* value < 0.05 was considered statistically significant.

## 3. Results

### 3.1. The Mortality of Rats

Mortality rate was low in rats following TBI. Two rats died during the experiments. One rat was in the TBI model group, and the other was in the calcitriol group.

### 3.2. Treatment of Calcitriol Attenuated Neurological Deficits

**Figure 1:**
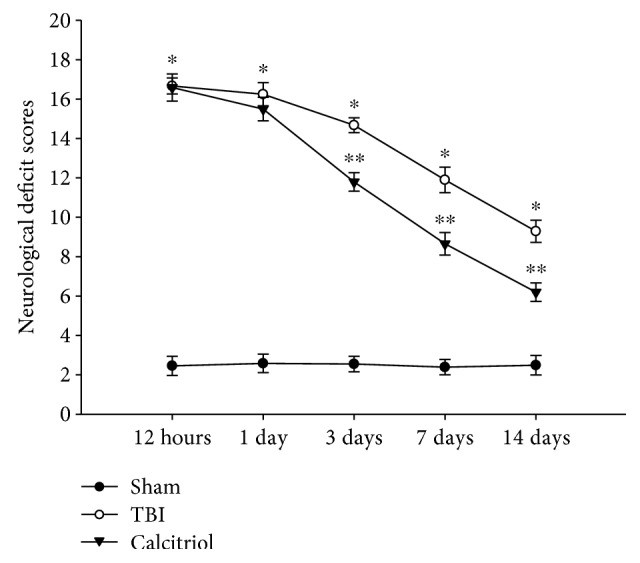
The effect of calcitriol on TBI-induced neurological deficits. The time course variation of neurological deficits was determined by neurological severity score tests. Dates represent mean ± standard error (*n* = 8, per group). ^∗^*P* < 0.01 versus the sham group; ^∗∗^*P* < 0.01 versus the TBI group.

The neurological severity scores were observed at 12 h to 14 days after TBI or sham operation. Compared with sham-operated animals, the neurological injury was significantly increased in the TBI group (*P* < 0.01). Nevertheless, calcitriol significantly reduced neurological deficits of rats at 3, 7, and 14 days (*P* < 0.01 versus the TBI group) (Figure 1). These observations indicated that the treatment of calcitriol improved neurological behavior after TBI.

### 3.3. Treatment of Calcitriol Attenuated the Brain Edema

**Figure 2:**
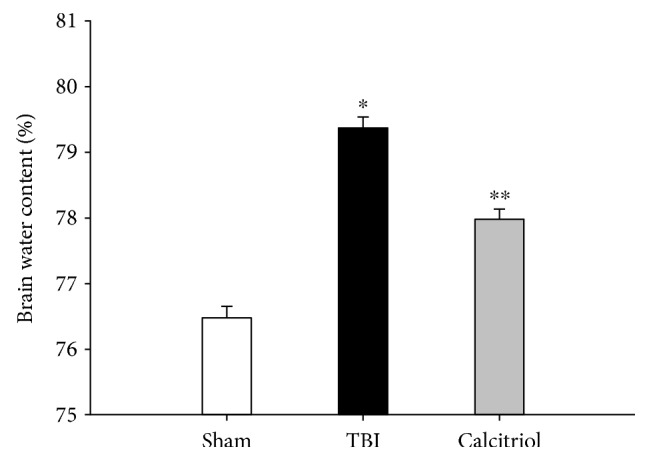
The effect of calcitriol on TBI-induced brain edema. The cerebral water content of rats was analyzed 3 days post-TBI or sham operation. Bars represent mean ± standard error (*n* = 5, per group). ^∗^*P* < 0.01 versus the sham group; ^∗∗^*P* < 0.01 versus the TBI group.

To identify the effect of calcitriol on cerebral edema, the analysis of brain water content was conducted at 3 days post-TBI or sham operation. Compared with the sham group, ICH caused a remarkable increase of brain water content (*P* < 0.01). And the brain water content of rat brains was significantly attenuated in the calcitriol group (*P* < 0.01 versus the TBI group) (Figure 2). These results indicated that calcitriol attenuated cerebral edema after TBI in rats.

### 3.4. Treatment of Calcitriol Improved the Learning and Memory Ability

**Figure 3:**
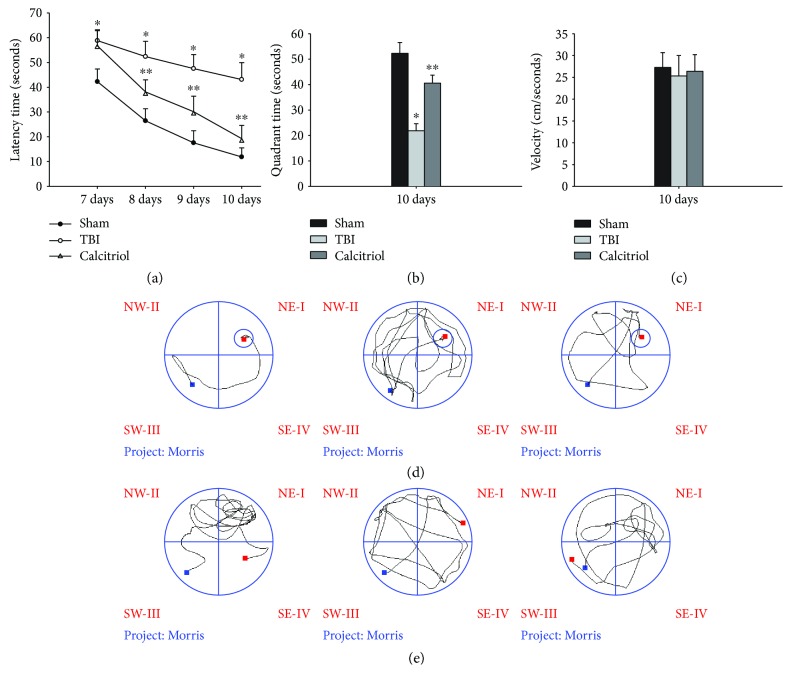
The effect of calcitriol on TBI-induced spatial memory deficits. Hippocampus-dependent cognitive capacity was evaluated using the MWM hidden platform task at 7–10 days post-TBI or sham operation. (a) Time (seconds) spent in finding the submerged platform at 7–10 days. (b) Exploration time (seconds) spent in the quadrant which initially contained the platform at 10 days. (c) There were no significant differences in swim speeds among groups. Representative traces indicating the sample paths of the rats from the maze latency trials (d) and the probe trials (e) on 10 days. Data are expressed as mean ± standard error (*n* = 7, per group). ^∗^*P* < 0.01 versus the sham group; ^∗∗^*P* < 0.01 versus the TBI group.

We next investigated whether calcitriol administration could improve the spatial memory deficits induced following TBI. Hippocampus-dependent cognitive capacity was evaluated using the MWM hidden platform task at 7–10 days post-TBI or sham operation.  Figure 3(a) illustrates the effects of calcitriol treatment on learning and memory ability during latency trials. TBI rats spent a longer time searching for the hidden platform at 7–10 days postsurgery (*P* < 0.01 versus the sham group). However, rats in the calcitriol group displayed a profoundly shorter latency time at 8–10 days as compared to those in the TBI group (*P* < 0.01). Representative trace diagrams indicating the latency time to finding the submerged platform at 10 days are depicted in Figure 3(d). In probe trails characterized by the removal of the hidden platform (Figure 3(b)), TBI rats displayed a worse learned bias navigating towards the goal quadrant, which previously contained the platform. They spent less time in the goal quadrant than their sham counterparts (*P* < 0.01). Calcitriol-treated rats, on the other hand, displayed improved learned bias, as evidenced by spending more time in the goal quadrant (*P* < 0.05 versus the TBI group). Representative traces obtained during the specified probed trials are depicted in Figure 3(e). Nevertheless, there were no significant differences in swim speeds among groups, indicating that the observed differences were not a result of the inability to execute the swim task (Figure 3(c)).

### 3.5. Treatment of Calcitriol Induced the Expression of VDR

**Figure 4:**
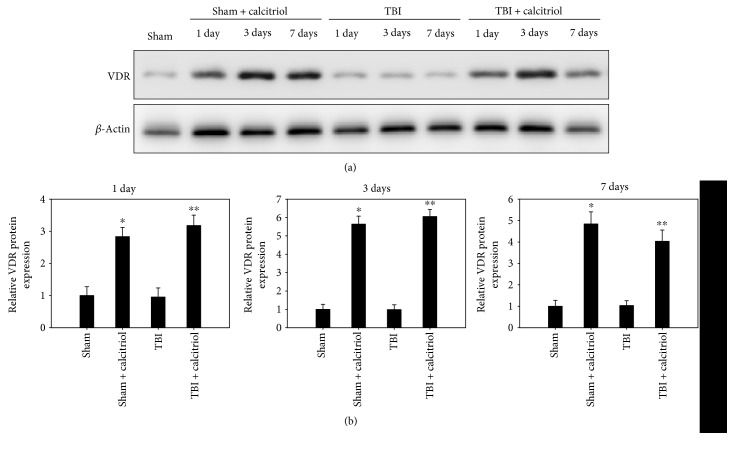
The effect of calcitriol on VDR protein expression. Western blot was performed to detect the expression of VDR at 1, 3, and 7 days in TBI or sham-operated rats. Densitometry analysis of VDR band was corresponding to *β*-actin. Bars represent mean ± standard error (*n* = 5, per time point). ^∗^*P* < 0.01 versus the sham group; ^∗∗^*P* < 0.01 versus the TBI group.

Western blot was performed to detect the expression of VDR protein at 1, 3, and 7 days in TBI or sham-operated rats. There was no significant difference in the expression of GAP-43 among the sham and TBI groups. Treatment of calcitriol significantly elevated the expression of VDR protein levels at 1, 3, and 7 days compared with the TBI group (*P* < 0.05) (Figure 4). These results indicated that calcitriol induced VDR expression in the hippocampus CA1 region of TBI rats.

### 3.6. Treatment of Calcitriol Attenuated NOX Activity and Expression of NOX_2_

**Figure 5:**
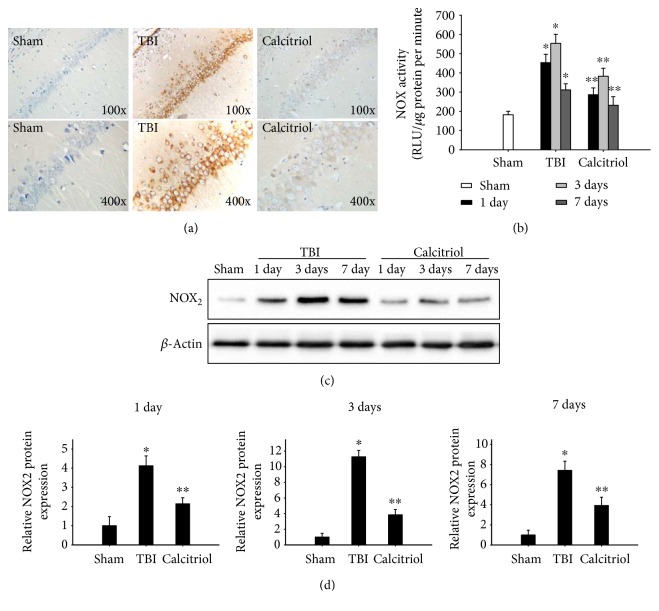
The effect of calcitriol on NOX activity and NOX_2_ protein expression. (a) Representative IHC staining of NOX_2_ in the hippocampus CA1 region from the sham, TBI, and calcitriol-treated groups at 3 days (*n* = 5, per group). (b) NOX activity was detected via a colorimetric assay at 1, 3, and 7 days in TBI or sham-operated rats. Bars represent mean ± standard error (*n* = 5, per time point). (c) Western blot analysis of NOX_2_ bands in the hippocampus CA1 region at 1, 3, and 7 days following TBI or sham surgery. (d) Densitometry analysis of NOX_2_ band corresponding to *β*-actin. Bars represent mean ± standard error (*n* = 5, per time point). ^∗^*P* < 0.01 versus the sham group; ^∗∗^*P* < 0.01 versus the TBI group.

IHC staining was evaluated to examine the role of the major NOX_2_ isoform of NADPH oxidase at 3 days after TBI or sham operation. As shown in Figure 5(a), we could occasionally observe positive cells, and the positive cells were lightly stained in the sham group. Obviously, NOX_2_-positive cells were widely distributed in the TBI group, staining with a deep color and indicating enhanced immune reactivity. However, the immune reactivity of NOX_2_ in the calcitriol group was weaker than that in the TBI group. We then performed a colorimetric assay to determine whether calcitriol treatment reduced NOX activity. As demonstrated in Figure 5(b), a marked elevation of NOX activity was observed at 1, 3, and 7 days in the hippocampus CA1 region following TBI induction (*P* < 0.01 versus the sham group). Calcitriol treatment significantly attenuated NOX activity compared with the TBI group (*P* < 0.01). Afterwards, western blot was performed to detect the expression of NOX_2_ in the hippocampus CA1 region in TBI or sham-operated rats (Figure 5(c)). As demonstrated by densitometry analysis in Figure 5(d), the NOX_2_ protein was expressed at low levels in the sham group. Following TBI, NOX_2_ levels were markedly increased at 1, 3, and 7 days (*P* < 0.01 versus the sham group). Calcitriol-treated rats, on the other hand, displayed reduced expression of NOX_2_ at 1, 3, and 7 days compared with the TBI group (*P* < 0.01).

### 3.7. Treatment of Calcitriol Improved Neuronal Survival in Hippocampus CA1 Region

**Figure 6:**
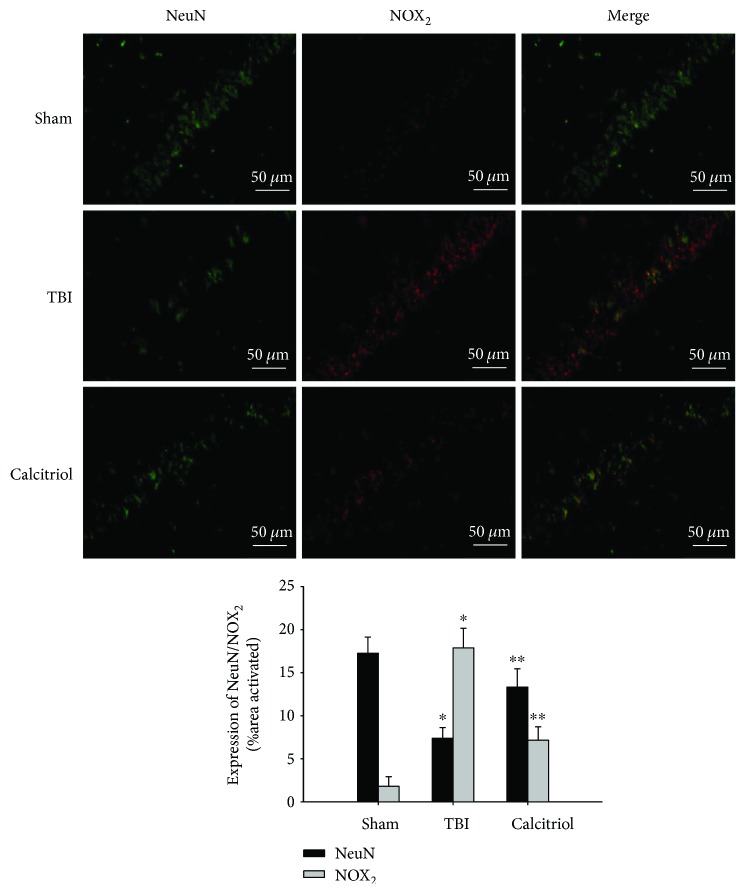
Double immunofluorescent staining of NOX_2_ and NeuN. Representative confocal images stained for NOX_2_ (red) and NeuN (green) showing calcitriol treatment not only reduced protein expression of NOX_2_ but it also markedly increased neuronal survival (scale bar, 50 *μ*m). Quantification of fluorescence intensity was analyzed using MATLAB software. Bars represent mean ± standard error (*n* = 5, per group). ^∗^*P* < 0.01 versus the sham group; ^∗∗^*P* < 0.01 versus the TBI group.

To further clarify the roles of NOX_2_ in the process of TBI-induced neuronal death, the colocalization of NeuN and NOX_2_ was assessed by double immunofluorescence staining at 3 days after TBI or sham operation. As shown in Figure 6, staining for NeuN and NOX_2_ revealed that TBI induced a profound loss of NeuN staining with an elevation of NOX_2_ staining in the hippocampus CA1 region as compared to the sham group (*P* < 0.01). Nevertheless, calcitriol treatment strongly suppressed the elevation of NOX_2_, whereas it increased the staining of NeuN (*P* < 0.01 versus the TBI group). These results indicated that calcitriol not only attenuated the expression of NOX_2_ in the hippocampus CA1 region but also exerted a robust neuroprotective effect against neuronal death.

### 3.8. Treatment of Calcitriol Suppressed Apoptosis in Hippocampus CA1 Region

**Figure 7:**
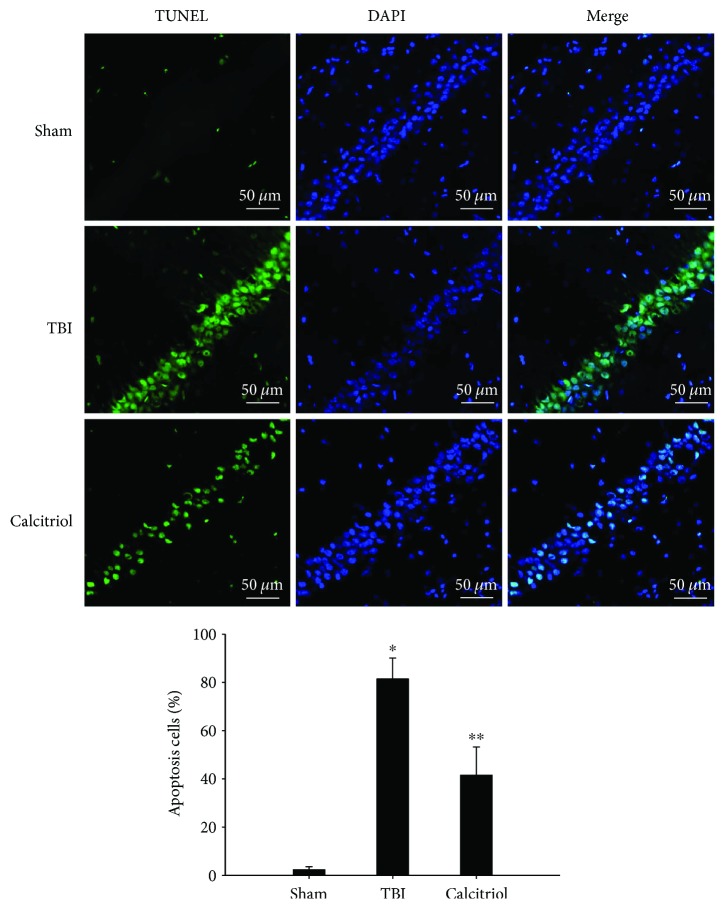
The effect of calcitriol on TBI-induced cell apoptosis. Apoptotic cell death was assessed by DAPI and TUNEL staining at 3 days (scale bar, 50 *μ*m). Representative confocal images stained for TUNEL (green) and DAPI (blue) showed the effect of calcitriol on TBI-induced cell apoptosis. Bars represent statistical analysis of relative apoptotic cell rate. Data are presented as the mean ± standard error (*n* = 5, per group). ^∗^*P* < 0.01 versus the sham group; ^∗∗^*P* < 0.01 versus the TBI group.

**Figure 8:**
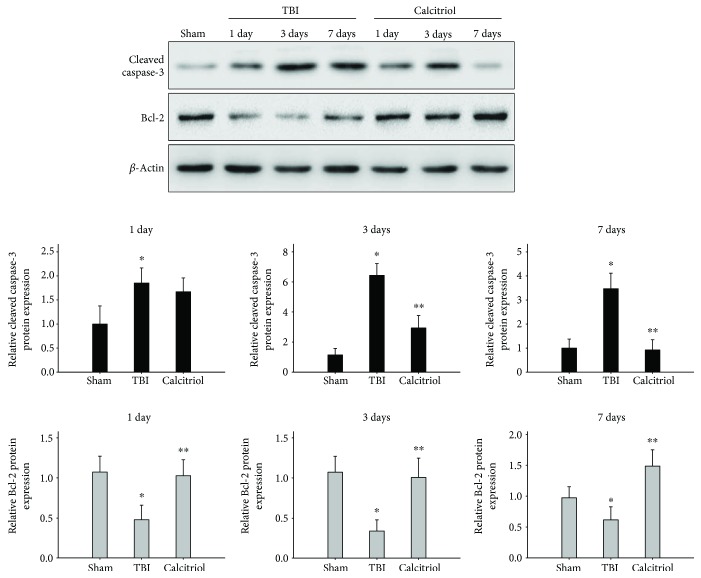
The effect of calcitriol on cleaved caspase-3 and Bcl-2 protein expression. Western blot was performed to detect the expression of cleaved caspase-3 and Bcl-2 at 1, 3, and 7 days in TBI or sham-operated rats. Densitometry analysis of cleaved caspase-3 and Bcl-2 band was corresponded to *β*-actin. Bars represent mean ± standard error (*n* = 5, per time point). ^∗^*P* < 0.01 versus the sham group; ^∗∗^*P* < 0.01 versus the TBI group.

To evaluate the ability of calcitriol to inhibit apoptosis, we first used the TUNEL method at 3 days after TBI or sham operation. Our results indicated that apoptosis cell rate in the hippocampus CA1 region was remarkably increased after TBI (*P* < 0.01 versus the sham group). But the rate of apoptotic cells in the calcitriol group was significantly reduced compared to that observed in the TBI group (*P* < 0.01) (Figure 7). Additionally, the protein levels of cleaved caspase-3 and Bcl-2 were detected by western blot at 1, 3, and 7 days in TBI or sham-operated rats. As shown in Figure 8, TBI increased cleaved caspase-3 expression whereas attenuated Bcl-2 expression compared to the sham-operated rats at 1, 3, and 7 days (*P* < 0.01). And the levels of cleaved caspase-3 were significantly decreased in the calcitriol group at 3 and 7 days (*P* < 0.01 versus the TBI group). Otherwise, calcitriol dramatically elevated Bcl-2 expression at 1, 3, and 7 days (*P* < 0.01 versus the TBI group). These results indicated that calcitriol administration effectively suppressed apoptosis in the hippocampus CA1 region post-TBI.

### 3.9. Treatment of Calcitriol Attenuated NOX_2_ Expression in Neurons

**Figure 9:**
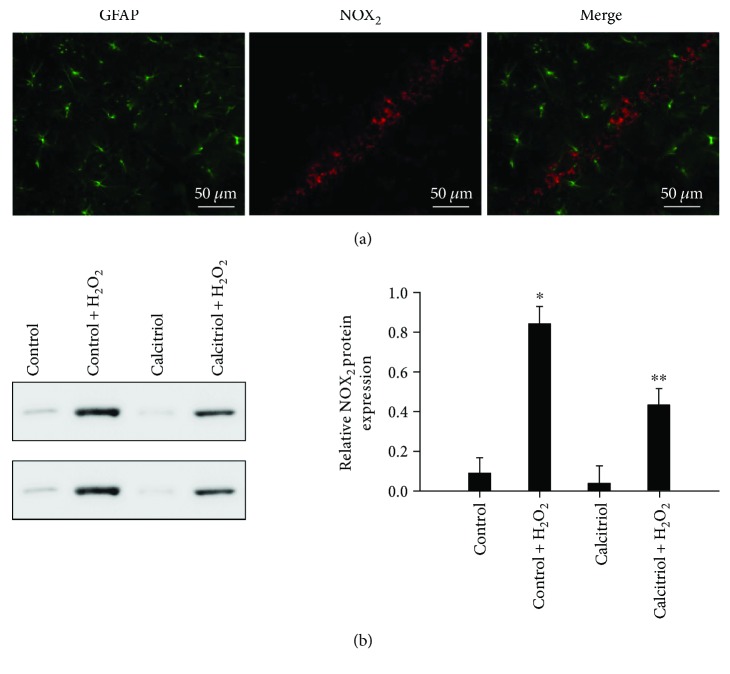
Calcitriol affects NOX_2_ expression in neurons. (a) Double immunofluorescent staining of NOX_2_ and GFAP. NOX_2_ and GFAP do not show colocalization. (b) Western blot analysis of NOX_2_ bands in HT22 cells. Densitometry analysis of NOX_2_ band corresponding to *β*-actin. Bars represent mean ± standard error (*n* = 5). ^∗^*P* < 0.01 versus the control group; ^∗∗^*P* < 0.01 versus the control + H_2_O_2_ group.

To determine the type of cells with elevated NOX_2_ after TBI, the colocalization of NOX_2_ and neurons/glial cells was assessed by double immunofluorescence staining. As shown in Figure 6, the colocalization of NeuN and NOX_2_ revealed that the elevated NOX_2_ after TBI was in neurons. Otherwise, NOX_2_ and GFAP do not show colocalization (Figure 9(a)). As the cell type specificity for NOX_2_ is determined, additional in vitro cell culture experiment should be performed to measure whether calcitriol affects NOX_2_ activity or expression in those cells. HT22 cells, an immortalized mouse hippocampus cell line, are used in vitro for mechanistic studies related to oxidative stress-induced cell death [30]. As demonstrated in Figure 9(b), exposure to H_2_O_2_ markedly increased NOX_2_ levels in HT22 cells (*P* < 0.01 versus the control group). Calcitriol-treated cells, on the other hand, displayed reduced expression of NOX_2_ compared with the control + H_2_O_2_ group (*P* < 0.01). These results indicated that calcitriol administration attenuated NOX_2_ expression in neurons.

## 4. Discussion

It is well known that TBI can result in significant neurological and cognitive deficits in humans [3]. The purpose of the current study was to assess the neuroprotective effects of calcitriol on TBI. We observed that TBI-induced neurological deficits were suppressed by calcitriol treatment. And calcitriol also improved the learning and memory ability of TBI rats. Traumatic brain edema impairs cerebral perfusion and oxygenation and increases intracranial pressure, leading to an expansion of brain volume which has a considerable influence on morbidity and mortality following TBI [31]. In the past years, numerous studies proved that traumatically injured tissue releases substances which enhance both cytotoxic and vasogenic brain edemas. In particular, such mediators include Ca^+^ ions, K^+^ ions, H^+^ ions, glutamate, histamine, and oxygen free radicals [32]. Nowadays, therapy of traumatic brain edema is still mainly symptomatic because all treatment styles used are directed decreasing intracranial pressure [31]. It still lacks a potent drug to attenuate traumatic brain edema formation, and it is progressing to date. Since our previous experiments showed that cerebral edema reached a peak at 3 days after injury [8, 24, 29], the water content of the brain tissue was measured at 3 days in TBI or sham-operated rats in this study. We found that calcitriol treatment significantly reduced cerebral edema induced after TBI. Taken together, above results were consistent with a previous study that calcitriol could exert neuroprotection in various models [16–19]. We hypothesize that calcitriol has the potential to become a novel therapeutics in treating TBI patients.

VD is most commonly associated with the regulation of calcium homeostasis [33]. VD2 and VD3 are two exogenous forms of VD, both of which are biologically inert. The activation of them requires two-step hydroxylation reaction involving 25-hydroxylase in the liver and 1*α*-hydroxylase in the kidney [34]. The biologically active metabolite of VD (calcitriol) exerts its endocrinological influence via a nuclear VDR [35]. The wide distribution of VDR suggests that vitamin D may regulate various physiological pathways, such as brain development, inflammation, neurological function, cell cycle control, and immune modulation and apoptosis [11–15]. In the present study, at the molecular levels, VDR expression in the hippocampus CA1 region was significantly elevated following calcitriol treatment. Firstly, VDR activation suppressed intracellular Ca^2+^ through increased intracellular Ca^2+^ buffering and decreased L-type voltage-sensitive Ca^2+^ channels, which caused a reduction of indiscriminate glutamate release and resultant neurotoxicity [36, 37]. In particular, cell apoptosis could be stimulated by the neuronal excitotoxic glutamate release and calcium influx after trauma [38, 39]. In line with previous studies, we also observed that calcitriol administration suppressed the high cell apoptosis rate in the hippocampus CA1 region induced following TBI. Secondly, Cekic and colleagues have found that activation of VDR attenuated the inflammatory response induced by TBI [38]. It has been demonstrated that a post-TBI reduction of neuroinflammation alleviated brain damage and decreased neurons apoptosis as well as improved functional outcomes in previous studies [40, 41]. Thirdly, Kalueff and colleagues have shown that VDR activation upregulated free radical scavenging and downregulated oxidative stress [39]. And in vitro research has revealed that the antioxidative effect was related to increasing expression of intracellular glutathione [42]. Lastly, activation of VDR could also enhance microtubule and neuronal cytoskeleton stability [38], promoting regeneration of the axons postinjury [43]. Thus, the mechanism of calcitriol neuroprotection is complex and multidimensional.

A novel finding here was that calcitriol treatment could inhibit the “activity” of NADPH oxidase post-TBI in rats. In the present study, we focused on the expression of NOX_2_ in the rat TBI model. We found that the effect of calcitriol was associated with attenuating NOX activity and expression of NOX_2_ post-TBI. This phenomenon reveals that NOX_2_-dependent NADPH oxidase activity was inhibited by calcitriol. Previous studies using NOX_2_ mutant knockout mice or the specific NOX_2_ inhibitor, gp91ds-tat, found that TBI damage to the brain was likewise significantly attenuated in NOX_2_ knockout mice or the gp91ds-tat group [44, 45]. NOX_2_ has been shown to be highly expressed in the hippocampus and contribute significantly to neuronal cell death and functional impairments after TBI [8, 46]. Therefore, we evaluated neuronal damage in the hippocampus CA1 region using immunostaining for NeuN. The results showed marked morphological changes and neuronal loss in the TBI group. Immunostaining results also revealed that NOX_2_ was highly expressed in the hippocampal CA1 region at 3 days after TBI. The results of western blot depicted that NOX_2_ protein expression was enhanced at 1–7 days following TBI induction. Treatment with calcitriol not only reduced protein expression of NOX_2_ but also markedly increased neuronal survival. These results indicate that following TBI, NOX_2_ activation is pivotal in the additional aggravation of secondary brain injury. Our findings are similar to those of Dong et al., who demonstrated that in vivo and in vitro activation of VDR with calcitriol improved endothelial function and thus prevented NADPH oxidase overproduction [47].

TBI secondary injury is delayed and is believed to result from a combination of pathological factors after trauma. Evidence implied that neuronal apoptosis plays an important role in the secondary injury of TBI [48]. Sequential activation of caspases, a family of proteases, plays a pivotal role in cellular apoptosis in the central nervous system. Apoptotic stimuli such as ischemic injury trigger the activation of initiator caspases and subsequently the caspase cascade, finally leading to apoptotic cell death [49]. Of the various subtypes of caspases, caspase-3 is the principal caspase involved in neuronal cell death [49]. Otherwise, Bcl-2 plays an important role in the process of oxidative stress-induced apoptosis [50]. An in vivo study by Tortosa et al. has demonstrated that Bcl-2 protected neurons against oxidant stress and apoptosis [51]. It has also been reported that Bcl-2 blocked the release of apoptotic factors from the mitochondria into the cytoplasm, maintaining mitochondrial integrity in vitro [52]. Our results show that calcitriol treatment resulted in decreased cleaved caspase-3 protein levels but increased the Bcl-2 protein levels in the hippocampus CA1 region, which ultimately inhibited the progression of apoptosis after TBI. Although these data provide further evidence for the protective effect of calcitriol against TBI, it should be noted that the chronic use of calcitriol may cause hypercalcemia and calcitriol toxicity is strongly dependent on the duration and frequency of administration. Therefore, while the present study chose the relatively high dose of calcitriol based on previous findings to protect the brain from acute injury, further studies should be cautious to the drug-induced hypercalcemia, especially concerning the long-term use.

## 5. Conclusion

The present study showed that the administration of calcitriol alleviated neurobehavioral deficits and brain edema following TBI. These effects may be generated through the activation of VDR expression and thereby downregulated NOX_2_ activity as well as suppressed neuronal apoptosis. Calcitriol may be promising as a protective intervention after TBI, and more study is warranted for its clinical testing in the future.
